# A Tool That Assesses the Evidence, Transparency, and Usability of Online Health Information: Development and Reliability Assessment

**DOI:** 10.2196/aging.9216

**Published:** 2018-05-07

**Authors:** Maureen Dobbins, Susannah Watson, Kristin Read, Kelly Graham, Reza Yousefi Nooraie, Anthony J Levinson

**Affiliations:** ^1^ The National Collaborating Centre for Methods and Tools School of Nursing McMaster University Hamilton, ON Canada; ^2^ Health Evidence™ School of Nursing McMaster University Hamilton, ON Canada; ^3^ Institute of Health Policy, Management, and Evaluation University of Toronto Toronto, ON Canada; ^4^ Division of e-Learning Innovation McMaster University Hamilton, ON Canada

**Keywords:** knowledge translation, Consumer Health Information, consumer health standards, internet standards, Patient Education as Topic, Patient Education as standards, critical appraisal, online health information, reliability analysis

## Abstract

**Background:**

The internet is commonly used by older adults to obtain health information and this trend has markedly increased in the past decade. However, studies illustrate that much of the available online health information is not informed by good quality evidence, developed in a transparent way, or easy to use. Furthermore, studies highlight that the general public lacks the skills necessary to distinguish between online products that are credible and trustworthy and those that are not. A number of tools have been developed to assess the evidence, transparency, and usability of online health information; however, many have not been assessed for reliability or ease of use.

**Objective:**

The first objective of this study was to determine if a tool assessing the evidence, transparency, and usability of online health information exists that is easy and quick to use and has good reliability. No such tool was identified, so the second objective was to develop such a tool and assess it for reliability when used to assess online health information on topics of relevant to optimal aging.

**Methods:**

An electronic database search was conducted between 2002 and 2012 to identify published papers describing tools that assessed the evidence, transparency, and usability of online health information. Papers were retained if the tool described was assessed for reliability, assessed the quality of evidence used to create online health information, and was quick and easy to use. When no one tool met expectations, a new instrument was developed and tested for reliability. Reliability between two raters was assessed using the intraclass correlation coefficient (ICC) for each item at two time points. SPSS Statistics 22 software was used for statistical analyses and a one-way random effects model was used to report the results. The overall ICC was assessed for the instrument as a whole in July 2015. The threshold for retaining items was ICC>0.60 (ie, “good” reliability).

**Results:**

All tools identified that evaluated online health information were either too complex, took a long time to complete, had poor reliability, or had not undergone reliability assessment. A new instrument was developed and assessed for reliability in April 2014. Three items had an ICC<0.60 (ie, “good” reliability). One of these items was removed (“minimal scrolling”) and two were retained but reworded for clarity. Four new items were added that assessed the level of research evidence that informed the online health information and the tool was retested in July 2015. The total ICC score showed excellent agreement with both single measures (ICC=0.988; CI 0.982–0.992) and average measures (ICC=0.994; CI 0.991–0.996).

**Conclusions:**

The results of this study suggest that this new tool is reliable for assessing the evidence, transparency, and usability of online health information that is relevant to optimal aging.

## Introduction

Many people increasingly turn to the internet as a source of information, motivation, and support for healthy living and management of common health conditions [1,2], including many older adults (those 60 years or older) [3]. At least half of the older adults who use the internet search for online medical or health-related information [4], and many of those who do not use the internet themselves have friends, family, and informal caregivers who seek online information on their behalf [2,5]. The availability of online health information has been shown to aid self-management of health conditions, particularly if the information can be tailored to the patient’s needs and/or paired with support [6-8].

Furthermore, access to online health information can help people stay up to date with emerging information about their health conditions and can facilitate shared decision-making between patients and health care providers [9,10]. However, for online health information to be helpful for patients it needs to be evidence-based. For online health information to be evidence-based, it should be based upon evidence that has been systematically and scientifically obtained [11]. Studies show, however, that much of the online health information accessed by the general public has not been informed by good quality evidence [12-18] and is therefore unlikely to produce the purported health benefits. Finally, studies show that the general public lacks the skills necessary to distinguish evidence-based resources from those that are not [19-22].

As Khazaal et al [23] noted, “content quality indicators are warranted in order to help patients and consumers judge the quality of online information and to discriminate good sites from others.” As a result, a number of tools have been developed to assess the extent to which evidence has been used in developing online health information. Some of these tools have even undergone psychometric testing. In 1999, Kim et al [24] identified 29 published rating tools and extracted 165 explicit criteria which they grouped into 13 distinct categories. The categories ranged from content (30% of criteria) to authority (11% of criteria) to user support (2% of criteria) [24]. In 2002 a review by Eysenbach et al [19] identified 86 unique quality criteria among 79 studies evaluating the quality of websites. The authors reduced these to the 22 most commonly-used criteria and concluded that operational definitions of the criteria were needed. In 2005 Bernstam et al [25] published a paper of operational definitions for these 22 criteria and reported that interrater reliability for 18 of the 22 items was good when precise operational definitions were provided. However, Bernstam et al [25] also noted that for some criteria, even when precise operational definitions existed, they could not be reliably assessed.

In yet another review by Gagliardi and Jadad published in 2002 [26], 98 “award-like” instruments used to rate the quality of online health information were identified. “Award-like” instruments take the form of logos or “seals of approval.” Only 11 of the 98 instruments provided information by which they could be evaluated, and none had been validated [26]. The 11 instruments were assessed against three criteria judged to be indicators of accurate online information (authorship, attribution, and disclosure), of which only three contained all three indicators of accuracy, and none which had been tested for reliability [26].

In 2005 Bernstam et al [27] published another review of tools to assess the quality of health information that could be used by patients. To be included in the review the tool had to be: (1) available to consumers, (2) made up of a limited number of items (10 or fewer), (3) made up of items that were objective and therefore assessable by consumers, and (4) readable. A total of 273 unique tools were identified; however, only 21 had 10 or fewer items, of which only 7 were made up of entirely objective items, with only one readable at a grade 8 reading level (which is no longer available).

In 2006 Provost et al [28] conducted a review of the literature to identify constructs thought to indicate quality of online health information, with the aim of developing a new instrument to assess the quality of health-related websites. The authors employed the 13 categories identified by Kim et al [24] and categorized 384 items identified through their literature review to these 13 categories. The authors eliminated criteria through discussion regarding 3 aspects of feasibility: (1) *externality*, being feasible to answer the question with the information available on the website; (2) *expertise independent*, being feasible to answer by the intended user of the scale independently of their credentials; and (3) *timeliness*, time efficiency in assessing the item. The study convened a panel of six experts to assess the items for relevance, importance, clarity, and feasibility [24]. The result was a new tool called the WebMedQual scale comprised of 8 categories, 8 subcategories, 95 items, and 3 supplemental items [24]. However, the tool was not tested for reliability.

Finally, Breckons et al [29] published a review in 2008 comparing 12 instruments that were used to assess the quality of complementary medicine information on the internet. The instruments contained between 4 and 43 items and varied considerably on what they assessed and how easy they were to use. While there was good agreement across the 12 instruments in the rank order of the assessed websites, only two of the instruments had been tested for reliability.

Clearly, a considerable amount of effort has been invested in the development of tools to assess the quality of online health information. However, it is not yet clear if there is one tool that is superior to all others with respect to being quick and easy to use and that reliably determines the quality of online health information. Furthermore, while quality assessment tools may help older adults more easily identify evidence-based information, a potentially more effective service might be one that compiles available online health information in one place, and assesses its quality. In particular, gateways or portals have been deemed particularly useful as they provide access to content that has been prescreened and deemed of high enough quality to be approved by a governing organization [29].

The McMaster Optimal Aging Portal (the Portal), launched in 2014, is a health information website that serves as such a gateway, providing access to online resources about healthy aging that have been preappraised for quality [30-32]. Healthy aging is, “a lifelong process of optimizing opportunities for improving and preserving health and physical, social, and mental wellness, independence, quality of life, and enhancing successful life-course transitions” [33]. The Portal offers direct and easy access to evidence-based information about how to stay healthy, active, and engaged, and how to manage health conditions as one grows older. Web Resource Ratings are one type of knowledge product available on the Portal. For the purposes of the Portal, a Web resource (online health information) is any item found online that can be read, watched, listened to, or interacted with (eg, fact sheets, webpages, and videos). The aim of the Web Resource Rating function is to assess the quality of online health information, to convert these assessments into star-ratings, and to post the star-ratings for individual online health information products on the Portal. The overarching goal is to help older adults easily identify and link to the highest quality online health information. The ability to complete this function on the Portal is dependent on the existence of a reliable quality assessment tool that is both easy and quick to use. The purpose of this study was to determine if there was at least one tool in existence with proven reliability that was quick and easy to use. If no such tool was identified, efforts would then be directed toward developing a new tool that would be quick and easy to use, followed by testing the new tool for reliability.

## Methods

### Identification of Articles Describing Instruments

A search for instruments that assessed the quality of online health information was conducted through an electronic search of Medline from 2002 and 2012, a focused internet search, and through suggestions made by key informants. The search strategy used is described in Multimedia Appendix 1. Title and abstract screening occurred with articles meeting the following inclusion criteria being retained for further assessment: an evaluation of an instrument assessing the quality of online information was reported, or it was a literature review of instruments assessing the quality of online information. Articles were excluded if: the focus was a health condition-specific website or tool, the instrument was only assessed for readability, or the instrument was physician-centered.

### Assessment of Relevance of Unique Instruments

Relevant articles underwent a second relevance assessment to identify instruments within those articles that: (1) had been assessed for reliability, (2) assessed the quality of the evidence used to create online information, (3) had fewer than 15 criteria, and (4) were suitable for use by citizen raters.

### Relevance Assessors

Assessments were independently completed by two raters. All raters had achieved (or were in the final year of) an undergraduate degree at McMaster University, had been working with the Portal for 5-10 hours per week for 1-6 months, and received training from the project coordinator (SW).

### Identification of Time to Complete Each Instrument and its Ease of use

Instruments retained from the second relevance assessment were then used to assess a sample of online health resources. Raters took note of how long it took to complete assessments for each instrument as well as how complex items within each instrument were to apply. Agreement between raters was assessed and the Portal team met to decide which instruments, if any, were appropriate for the purposes of the Portal. Assessments were completed by dyads with one assessor being a staff member (as described above for relevance assessment), and the second being a Lead of the Portal (MD, BH, JL; each of whom have decades of experience in evidence-based practice and appraisal of evidence) [31].

### Development of a new Instrument

No one tool was deemed sufficient for its intended use for the Portal, so the development of a new instrument was begun. Items for the new instrument were crafted either anew by the Portal team or selected from the previously identified instruments. Items were developed and/or selected to meet the following expectations: (1) the answer needed to be dichotomous (Yes or No); (2) the items were suitable for assessing a Web resource on a website, rather than a website; (3) the information needed to assess the item would reasonably be included on the webpage of the resource; (4) had good reliability; and (5) was suitable for use by citizen raters. The items were organized into the following three categories: (1) the quality of the evidence which informed the Web resource, (2) the transparency of the resource development process, and (3) the usability of the resource. A guidance document explaining each item and how it should be rated was created and used to train raters, and was used as a resource while raters completed their assessments.

### Reliability Assessment

A set of 10 items was formally assessed for reliability in April 2014 using 120 Web resources relevant to healthy aging (2 raters, therefore a total of 240 ratings), with a second reliability assessment being conducted in July 2015 using a different set of 107 Web resources (214 ratings). The Portal used in this study employs a two-stage process for identifying and selecting Web resources. These tasks were completed by the same staff as described above for relevance assessment. In stage 1 internet searches are conducted to identify websites (worldwide) providing information relevant to healthy aging (ie, physical activity, nutrition, social engagement). Websites are assessed for the following criteria: the website is not funded by a company trying to sell products or services, content of the site is relevant to healthy aging, the website includes content intended for use by citizens, and the website is freely accessible. Websites meeting all of these criteria are deemed relevant, and move on to stage 2, which is identification and selection of Web resources housed on the website. Potentially relevant resources are uploaded to a content management system. Each Web resource is then assessed for the following: the resource is not funded by a company trying to sell products or services, the resource is relevant to healthy aging, the resource is intended for use by citizens, and the resource is less than 3 years old. Web resources meeting all four criteria then undergo quality assessment.

For this study a team of eight raters completed the quality assessments, with each Web resource being rated by two independent raters. Consistent with relevance assessment, all raters had achieved (or were in the final year of) an undergraduate degree at McMaster University and had been rating resources for 1-6 months part-time (5-10 hours per week). All raters received training on using the instrument. Ratings were conducted independently and conflicts were resolved through discussion. A third reviewer (MD or SW) resolved any conflicts in ratings. Data were exported in bulk from the online rating system into SPSS Statistics 22 software for statistical analyses.

Reliability between two raters for each item included in the instrument was assessed using the intraclass correlation coefficient (ICC). The ICC is defined as the correlation between one measurement on a target (in this case, the Web resource) and another rating on the same target [34]. Four value ranges, as outlined in McDowell [35], were used to interpret the ICCs: ICC values >0.75 were considered “excellent” reliability; values from 0.6 to 0.74 had “good” reliability; values from 0.4 to 0.59 had “fair” reliability; and values below 0.4 had “poor” reliability. The threshold for retaining items was >0.60 (“good” reliability).

ICC values were assessed for each individual item in both 2014 and July 2015. The overall ICC was assessed for the instrument as a whole in July 2015 once the final set of items was identified. A one-way random effects model was used to report the results; this model assumes that raters are randomly selected from a population of raters and different pairs of raters rate each product. Both the average and single measures were included in the analysis. Average measures calculate the mean reliability (selection of the same rating for the same criteria) of multiple raters. Single measures calculate the reliability of a single rater, accounting for any potential rater effect (ie, chance and error affecting variance in rater selections) [34].

## Results

### Findings From the Literature Search for Existing Instruments

Once duplicates were removed, 585 articles were identified, of which 19 were either an evaluation of an instrument assessing the quality of online information or a literature review of instruments assessing the quality of online information [23-29,36-47]. Among the 19 articles there were no instruments identified that met all of the following criteria: (1) had been assessed for reliability, (2) assessed the quality of the evidence used to create a Web resource, (3) had fewer than 15 criteria, and (4) were suitable for use by citizen raters. However, five instruments met two of the criteria: had been assessed for reliability and contained criteria that assessed the quality of the evidence used to create a Web resource. These five instruments were retained for further assessment. These instruments included the DISCERN instrument [48], the Information Quality Tool (IQT) [42], the Quality Scale (QS) [49], the Minervation validation instrument for healthcare websites (LIDA tool) [50], and a set of 22 criteria identified by Bernstam et al [25] as those most commonly used to assess the quality of online health information.

The DISCERN instrument is a 16-item instrument using a 5-point Likert scale rating system, which was developed by an expert panel to evaluate the reliability and quality of treatment information for a particular health problem [48]. The IQT is a 21-item instrument of yes or no questions about a resource’s authorship, sponsorship, currency, accuracy, confidentiality, and navigability. Criteria are weighted by importance and a total score is calculated that ranges from 0 to 4 [42]. The QS is a 7-item instrument using a 3-point Likert scale rating system. The total score can range from 0 to 14 and includes criteria related to ownership, authorship, source, currency, interactivity, navigability, and balance [49]. The LIDA Instrument developed by Minervation looks at three areas to evaluate online health information (accessibility, usability, and reliability) using a four-point scale ranging from *always* to *never*. There are 12 sub-subsets of questions and total scores are generated for each of the three sections [50]. In Bernstam et al [25], authors evaluated the interrater agreement of 22 common technical quality criteria. Criteria included questions related to specific webpages (eg, authorship, credentials, date, and references) as well as questions related to the general website (eg, internal search engine, feedback mechanism, and editorial review process). Use of the five instruments to assess a sample of Web resources by Portal dyads determined that they all took too long to complete, or were too complex to apply, and therefore a new instrument was developed with reliability being formally assessed in April 2014 on a set of 10 items.

**Table 1:** Reliability assessment of Web Resource Rating criteria measured by intraclass correlation coefficient, April 2014. n=120 resources/240 ratings.

Criteria		Intraclass Correlation Coefficient (95% CI)	
		Single measures	Average measures
**Evidence Base**			
	1. Does the product comment on the quality of the evidence?	0.929 (0.900-0.950)	0.963 (0.948-0.975)
	2. Does the product use language that communicates the strength of recommendation(s)?	0.548 (0.410-0.662)	0.708 (0.581-0.796)
**Transparency**			
	3. Are sources provided for each claim/recommendation?	0.728 (0.632-0.802)	0.843 (0.774-0.890)
	4. Authorship disclosure. Is the authors’ or editors’ name and affiliation disclosed?	0.465 (0.313-0.594)	0.635 (0.476-0.745)
	5. Is advertising clearly labelled?	0.838 (0.776-0.884)	0.912 (0.874-0.939)
	6. Is the date of creation within the last three years?	0.822 (0.754-0.872)	0.902 (0.860-0.932)
	7. Is there a feedback mechanism?	0.724 (0.627-0.799)	0.840 (0.771-0.888)
**Usability**				
	8. Minimal scrolling	0.489 (0.340-0.614)	0.657 (0.508-0.761)
	9. Logical flow	0.660 (0.547-0.750)	0.796 (0.707-0.857)
	10. Accessibility (For text content: *can text be resized or is there a screen reader?* For nontext content: *is a transcription available?)*	0.719 (0.620-0.795)	0.836 (0.765-0.886)

### Results of the Reliability of the new Instrument

The results are presented in Table 1. Using the data for single measures, seven items had ICCs >0.60: (1) *Does the product comment on the quality of the evidence?*; (2) *Are sources provided for each claim/recommendation?*; (3) *Is advertising clearly labeled?*; (4) *Is the date of creation within the last three years?*; (5) *Is there a feedback mechanism?*; (6) *Is there logical flow?*; and (7) *Is the text accessible?*

Of the three items with ICCs <0.60, one was removed from the instrument (*minimal scrolling*), and the other two (*language that communicates the strength of the recommendation* and *authorship*) were kept, as they were regarded as priority items and had been identified in other instruments as important criteria [25,42,49]. Modifications were made to the wording of these two items for clarity, as well as the seven with ICC values >0.60, and they were reassessed in July 2015. In addition, four new items were added at that time that assessed the level of research evidence the Web resource was informed by: (1) *Is the Web resource informed by published single studies?*; (2) *Is the Web resource informed by randomized controlled trials?*; (3) *Is the Web resource informed by systematic reviews/meta-analyses?*; and (4) *Is the Web resource informed by best practice guidelines?* Of this set of 13 items, six were related to the quality of the evidence, five were related to the transparency of the development of the Web resource, and two assessed usability.

The results of this reliability assessment illustrated that 11 of the 13 items had excellent ICC scores, and two (*Is the strength of the recommendations provided?* and *Are peer-reviewed sources provided for each claim or recommendation?*) had good ICCs (Table 2). Furthermore, six items had an ICC of 1. Given the results of this assessment, it was decided that no further testing of the tool was required, and these 13 items became the final set of items for the instrument.

The ICC of the total rating score for the 13 items, calculated with a one-way random model, has excellent reliability with both single measures (ICC=0.988; CI 0.982-0.992) and average measures (ICC=0.994; CI 0.991-0.996), as depicted in Table 2. These results indicate that the instrument is highly reliable, whether ratings are conducted by a single, independent rater or are averaged from the results of at least two raters, with only approximately 1% of the variance in Web resource ratings attributed to chance or other factors. The final version of the tool is included in Multimedia Appendix 2.

**Table 2:** Reliability assessment of Web Resource Rating criteria measured by intraclass correlation coefficient, July 2015. n=107 resources/214 ratings.

Criteria		Intraclass Correlation Coefficient (95% CI)	
		Single measures	Average measures
**Evidence Base**				
	1. Is the Web resource informed by published single studies?	0.933 (0.904-0.954)	0.965 (0.949-0.976)
	2. Is the Web resource informed by published randomized controlled trials?	1	1
	3. Is the Web resource informed by published systematic reviews/meta-analyses?	1	1
	4. Is the Web resource informed by best practice guidelines?	1	1
	5. Is the quality of the evidence reported?	0.945 (0.921-0.962)	0.972 (0.959-0.981)
	6. Is the strength of recommendations provided?	0.660 (0.538-0.755)	0.795 (0.700-0.860)
**Transparency**				
	7. Are peer-reviewed sources provided for each claim/recommendation?	0.740 (0.641-0.815)	0.851 (0.781-0.898)
	8. Is the author’s or editor’s name and affiliations disclosed?	0.942 (0.917-0.960)	0.970 (0.957-0.980)
	9. Is the advertising clearly labelled (or is there no advertising)?	1	1
	10. Has the Web resource been created or updated within the last 3 years?	0.926 (0.893-0.949)	0.961 (0.943-0.974)
	11. Is there a feedback mechanism?	1	1
**Usability**				
	12. Logical flow: is the information easy to follow?	1	1
	13. Accessibility: does the Web resource offer options to access the information? *Can text be resized or is there a screen reader? For nontext content, is a transcription or subtitle option available?*	0.944 (0.920-0.962)	0.971 (0.958-0.980)
Total Score		0.988 (0.982-0.992)	0.994 (0.991-0.996)

## Discussion

### Assessment Criteria for Online Health Information

The purpose of this study was to determine if at least one instrument with proven reliability existed that was quick and easy to use for the assessment of online health information. If no such instrument was identified, the focus then became the development of a new instrument that was quick and easy to use, and to test the instrument for reliability. Although various quality assessment instruments specific to online resources exist, it was determined through this study that all identified instruments either had poor reliability or had not been assessed for reliability, had too many criteria to make the tool easy to use, or were not suitable for use by citizen raters.

As a result, a new instrument was created that incorporated items from existing instruments, as well as the development of new criteria. Formal reliability assessment, undertaken between April 2014 and July 2015, resulted in the identification of the 13 items included in the final version of the new instrument. The ICC assessment showed that–as of July 2015–the final set of 13 items had good-to-excellent reliability (ICC=0.660 to 1.0). Criterion 6 (*Is the strength of recommendations provided?*) had the lowest level of reliability (ICC = 0.660).

The one criterion eliminated due to low ICC during the reliability assessment was *usability*. Previous evidence has found that usability criteria such as navigability and readability tend to be more subjective and have been shown by others to lead to low reliability scores [42,51]. This assessment adds support to previously published studies, as only two usability criteria had ICCs greater than 0.6 and were therefore retained in the final version of the instrument.

As a result of this analysis, the new instrument can be recommended as reliable for assessing the quality of online health information, whether rated by one or two raters. It is important to place the results of this analysis within the context of other instruments available to assess the quality of online health information; however, the majority of these instruments have not been assessed for reliability. As a result, our comparison to other instruments is limited to DISCERN [48], IQT [42], QS [49], LIDA [50], and the Bernstam et al assessment of the 22 most common criteria for assessing online information [25]. The level of interrater reliability is higher for the new instrument (ICC=0.988) than for IQT (ICC=0.543) [42], LIDA (ICC=0.611) [50], QS (ICC=0.796) [42], and DISCERN (ICC=0.823) [42]. Individual criteria for the DISCERN, IQT, and QS instruments were assessed using kappa (*k)* coefficients or weighted *k* coefficients, with results ranging from *poor* (ICC=0.102) to *perfect agreement* (ICC=1.0) [25,42]. The new instrument compares favorably with these results, with a higher range of ICCs for individual criteria (ICC=0.660 to 1.0) as well as consistently higher scores for comparable criteria. For example, the new instrument shows a range of ICC scores for criteria related to the use of evidence in Web resource content of *good*-to-*perfect* reliability (ICC=0.660-1.0), which is higher than the range of similar criteria within both the IQT (ICC=0.553-0.899) and DISCERN tools (ICC=0.102-0.541) [42].

### Limitations

The new instrument was developed, and assessed for reliability through this analysis, to assess the *quality* of online resources. However, it is important to note that the ratings of this instrument are weighted to value the use of research evidence over other components such as transparency and usability. Although this weighting reflects the priorities and purpose of the Portal (to increase access to evidence-based information about healthy aging), not all internet users may judge *quality* by the same standards. While citizens may value usability features (ie, website appeal, ease of use, accessible language, and lack of advertisements, pop-ups, and other interference [52,53]), multiple studies (including this one) have consistently reported low ICC scores for usability-related items, which supports the decision to include only two such items in the final set of items for the new instrument [42,51]. Future research is needed to establish the feasibility of validated methods for assessing usability of online resources, particularly those targeting older adults.

The data for this analysis came from ratings conducted by an established staff of trained raters. Although the ICC analysis takes into account the impact of untrained raters on assessments, ongoing analyses will be useful to verify this with a group of trainees or members of the public (eg, university student trainees contributing to the development of website content, including the rating of online Web resources).

Lastly, it is important to note that the new instrument assesses the process of resource development and not the accuracy of the information or congruency of the content with the latest high-quality evidence. In the development phase of this instrument, there was discussion about including criteria to rate the accuracy of online health information. However, our aim was to create a quality assessment instrument that was easy for anyone to use; an accuracy check requires subject matter expertise, and raters having access to the latest high-quality research and the ability to search, appraise, and interpret the messages of this research, which was deemed inappropriate for citizen raters. The final set of items included in the new instrument values the use of high-quality evidence in resource development as a proxy for measuring the quality of claims and recommendations included in the resource. This approach has been used by others with similar types of instruments [12]. Further assessment is needed to determine if this hypothesis is true.

### Implications

This analysis not only illustrates that the new instrument is a reliable tool for assessing the quality of the process for developing online health information, but also supports the decision to move to a one-rater system for assessing Web resources. A small staff of 3-4 raters independently rate resources to publish on the McMaster Optimal Aging Portal; this saves considerable time, costs, and human resources toward the production of this content. Other practical implications of this analysis include the potential for external raters (eg, health professionals or citizens) to use this instrument to independently assess or design their own high-quality online health information. Future plans include making a copyrighted version of the instrument publicly available and using the instrument and ratings to provide guidance in developing high-quality online health information with health organizations and developers of health information websites. This new quality assessment instrument was designed to have a broad application, be adaptable to assess the quality of online health information relevant to topics across the health care continuum, and is intended for multiple audiences.

### Conclusions

The instrument developed and assessed in this study has excellent interrater reliability for overall rating score and good-to-excellent reliability for individual rating criteria. The instrument can be recommended as highly reliable for the assessment of online health information.

## Appendix

Multimedia Appendix 1 Medline search output for web resource rating instruments.

Multimedia Appendix 2 Web resource rating tool.

## Abbreviations

ICC: intraclass correlation coefficient
IQT: Information Quality Tool
QS: Quality Scale

## References

[1] (2013). Statistics Canada.

[2] Fox S, Duggan M (2013). Pew Internet and American Life Project.

[3] (2010). Statistics Canada.

[4] Veenhof B, Timusk P (2014). Statistics Canada.

[5] Washington KT, Meadows SE, Elliott SG, Koopman RJ (2011). Information needs of informal caregivers of older adults with chronic health conditions. Patient Educ Couns.

[6] Schulz DN, Kremers SP, Vandelanotte C, van Adrichem MJ, Schneider F, Candel MJ, de Vries H (2014). Effects of a web-based tailored multiple-lifestyle intervention for adults: a two-year randomized controlled trial comparing sequential and simultaneous delivery modes. J Med Internet Res.

[7] Sawesi S, Rashrash M, Phalakornkule K, Carpenter JS, Jones JF (2016). The impact of information technology on patient engagement and health behavior change: a systematic review of the literature. JMIR Med Inform.

[8] Pearson M, Mattke S, Shaw R, Ridgely S, Wiseman S (2012). Agency for Healthcare Research and Quality.

[9] (2014). Pew Research Centre.

[10] Finkelstein J, Knight A, Marinopoulos S, Gibbons MC, Berger Z, Aboumatar H, Wilson RF, Lau BD, Sharma R, Bass EB (2012). Enabling patient-centered care through health information technology. Evid Rep Technol Assess (Full Rep).

[11] Brownson RC, Fielding JE, Green LW (2017). Building capacity for evidence-based public health: reconciling the pulls of practice and the push of research. Annu Rev Public Health.

[12] Fahy E, Hardikar R, Fox A, Mackay S (2014). Quality of patient health information on the Internet: reviewing a complex and evolving landscape. Australas Med J.

[13] Ow D, Wetherell D, Papa N, Bolton D, Lawrentschuk N (2015). Patients' perspectives of accessibility and digital delivery of factual content provided by official medical and surgical specialty society websites: a qualitative assessment. Interact J Med Res.

[14] Devine T, Broderick J, Harris LM, Wu H, Hilfiker SW (2016). Making quality health websites a national public health priority: toward quality standards. J Med Internet Res.

[15] Pérez-López FR, Pérez Roncero GR (2006). Assessing the content and quality of information on the treatment of postmenopausal osteoporosis on the World Wide Web. Gynecol Endocrinol.

[16] Elamin MB, Montori VM, Burneo J, Demaerschalk B, Jenkins M (2012). The hierarchy of evidence: from unsystematic clinical observations to systematic reviews. Neurology.

[17] Dentzer S (2009). Communicating medical news--pitfalls of health care journalism. N Engl J Med.

[18] Cullen TA (2013). Online health information: Shortcomings and challenges.

[19] Eysenbach G, Köhler C (2002). How do consumers search for and appraise health information on the world wide web? Qualitative study using focus groups, usability tests, and in-depth interviews. BMJ.

[20] Coulter A, Ellins J, Swain D, Clarke A, Heron P, Rasul F, Magee H, Sheldon H (2006). Assessing the quality of information to support people in making decisions about their health and healthcare.

[21] Silver MP (2015). Patient perspectives on online health information and communication with doctors: a qualitative study of patients 50 years old and over. J Med Internet Res.

[22] Manafo E, Wong S (2012). Exploring older adults' health information seeking behaviors. J Nutr Educ Behav.

[23] Khazaal Y, Chatton A, Zullino D, Khan R (2012). HON label and DISCERN as content quality indicators of health-related websites. Psychiatr Q.

[24] Kim P, Eng TR, Deering MJ, Maxfield A (1999). Published criteria for evaluating health related web sites: review. BMJ.

[25] Bernstam EV, Sagaram S, Walji M, Johnson CW, Meric-Bernstam F (2005). Usability of quality measures for online health information: can commonly used technical quality criteria be reliably assessed?. Int J Med Inform.

[26] Gagliardi A, Jadad AR (2002). Examination of instruments used to rate quality of health information on the Internet: chronicle of a voyage with an unclear destination. BMJ.

[27] Bernstam EV, Shelton DM, Walji M, Meric-Bernstam F (2005). Instruments to assess the quality of health information on the World Wide Web: what can our patients actually use?. Int J Med Inform.

[28] Provost M, Koompalum D, Dong D, Martin BC (2006). The initial development of the WebMedQual scale: domain assessment of the construct of quality of health web sites. Int J Med Inform.

[29] Breckons M, Jones R, Morris J, Richardson J (2008). What do evaluation instruments tell us about the quality of complementary medicine information on the Internet?. J Med Internet Res.

[30] Dobbins M, Haynes RB, Iorio A, Lavis JN, Levinson AJ (2016). User experiences of the McMaster optimal aging portal's evidence summaries and blog posts: usability study. JMIR Hum Factors.

[31] Barbara AM, Dobbins M, Haynes RB, Iorio A, Lavis JN, Raina P, Levinson AJ (2016). The McMaster Optimal Aging Portal: usability evaluation of a unique evidence-based health information website. JMIR Hum Factors.

[32] Barbara AM, Dobbins M, Brian HR, Iorio A, Lavis JN, Raina P, Levinson AJ (2017). McMaster Optimal Aging Portal: an evidence-based database for geriatrics-focused health professionals. BMC Res Notes.

[33] (2002). Health Canada.

[34] Shrout PE, Fleiss JL (1979). Intraclass correlations: uses in assessing rater reliability. Psychol Bull.

[35] McDowell I (2006). Measuring health: a guide to rating scales and questionnaires. 3rd edition.

[36] Cline RJ, Haynes KM (2001). Consumer health information seeking on the Internet: the state of the art. Health Educ Res.

[37] Risk A, Dzenowagis J (2001). Review of Internet health information quality initiatives. J Med Internet Res.

[38] Craigie M, Loader B, Burrows R, Muncer S (2002). Reliability of health information on the Internet: an examination of experts' ratings. J Med Internet Res.

[39] Eysenbach G, Powell J, Kuss O, Sa E (2002). Empirical studies assessing the quality of health information for consumers on the world wide web: a systematic review. JAMA.

[40] Fallis D, Frické M (2002). Indicators of accuracy of consumer health information on the Internet: a study of indicators relating to information for managing fever in children in the home. J Am Med Inform Assoc.

[41] Wilson P (2002). How to find the good and avoid the bad or ugly: a short guide to tools for rating quality of health information on the Internet. BMJ.

[42] Ademiluyi G, Rees CE, Sheard CE (2003). Evaluating the reliability and validity of three tools to assess the quality of health information on the Internet. Patient Educ Couns.

[43] Griffiths KM, Christensen H (2005). Website quality indicators for consumers. J Med Internet Res.

[44] Bernstam EV, Walji MF, Sagaram S, Sagaram D, Johnson CW, Meric-Bernstam F (2008). Commonly cited website quality criteria are not effective at identifying inaccurate online information about breast cancer. Cancer.

[45] Deshpande A, Jadad AR (2009). Trying to measure the quality of health information on the Internet: is it time to move on?. J Rheumatol.

[46] Clark EJ (2002). Health care web sites: are they reliable?. J Med Syst.

[47] Adams SA (2010). Revisiting the online health information reliability debate in the wake of “Web 2.0”: an inter-disciplinary literature and website review. Int J Med Inform.

[48] Charnock D, Shepperd S, Needham G, Gann R (1999). DISCERN: an instrument for judging the quality of written consumer health information on treatment choices. J Epidemiol Community Health.

[49] Sandvik H (1999). Health information and interaction on the internet: a survey of female urinary incontinence. BMJ.

[50] (2008). Minervation.

[51] Shedlosky-Shoemaker R, Sturm AC, Saleem M, Kelly KM (2009). Tools for assessing readability and quality of health-related Web sites. J Genet Couns.

[52] Tao D, LeRouge CM, Deckard G, De Leo G (2012). Consumer perspectives on quality attributes in evaluating health websites.

[53] Dubowicz A, Schulz PJ (2015). Medical information on the Internet: a tool for measuring consumer perception of quality aspects. Interact J Med Res.

