# Supplementary material for: A Tool That Assesses the Evidence, Transparency, and Usability of Online Health Information: Development and Reliability Assessment
Source: JMIR Aging. 2018 May 7;1(1):e3. doi: 10.2196/aging.9216 (PMC6715399; doi:10.2196/aging.9216)
Supplement: Multimedia Appendix 1 [file aging_v1i1e3_app1.pdf]

Multimedia Appendix 1: Medline search output for web resource rating instruments

Database: Ovid MEDLINE(R) without Revisions <1996 to June Week 5 2016> Search Strategy:

----

1 exp *Certification
2 exp *Computer Communication Networks
3 *Health Education
4 exp *Information Management
5 *Information Services
6 exp Internet
7 *Medical Informatics
8 exp *Quality Control
9 exp *Quality Indicators, Health Care
10 *"Quality of Health Care"
11 exp *Total Quality Management
12 1 or 2 or 3 or 4 or 5 or 6 or 7 or 8 or 9 or 10 or 11
13 exp Internet or web*
14 exp Online Systems or online
15 electronic resources
16 13 or 14 or 15
17 scale or *"Weights and Measures"
18 scheme
19 tool
20 measur*
21 (rate or rating)
22 criteria
23 checklist or Checklist
24 17 or 18 or 19 or 20 or 21 or 22 or 23
25 12 and 16 and 24
26 exp *Certification
27 exp *Computer Communication Networks
28 *Health Education
29 exp *Information Management
30 *Information Services
31 exp Internet
32 *Medical Informatics
33 exp *Quality Control
34 exp *Quality Indicators, Health Care
35 *"Quality of Health Care"
36 exp *Total Quality Management
37 26 or 27 or 28 or 29 or 30 or 31 or 32 or 33 or 34 or 35 or 36
38 exp Internet or web*
39 exp Online Systems or online
40 electronic resources
41 38 or 39 or 40
42 scale or *"Weights and Measures"
43 scheme
44 tool
45 measur*
46 (rate or rating)
47 criteria
48 checklist or Checklist
49 42 or 43 or 44 or 45 or 46 or 47 or 48
50 37 and 41 and 49
