# Supplementary material for: A Tool That Assesses the Evidence, Transparency, and Usability of Online Health Information: Development and Reliability Assessment
Source: JMIR Aging. 2018 May 7;1(1):e3. doi: 10.2196/aging.9216 (PMC6715399; doi:10.2196/aging.9216)
Supplement: Multimedia Appendix 2 [file aging_v1i1e3_app2.pdf]

## Multimedia Appendix 2: The Web Resource Rating (WRR) Tool

**Web Resource Rating (WRR) tool**

*Intended for quality assessment of online web resources which are:*

- *Not directly funded by a company trying to sell a product or service*
- *Less than five years old*

| **Criteria** | **Yes (1*)** | **No (0)** |
|---|---|---|
| Evidence Base | | |
| **1. Is the web resource informed by published single studies?**<br>*Resource includes reference to original research such as published single studies from peer-reviewed sources, statistics, textbooks* | | |
| **2. Is the web resource informed by published randomized controlled trials (RCTs)?**<br>*Resource includes reference to RCTs in text or in a reference list* | | |
| **3. Is the web resource informed by published systematic reviews/meta analyses?**<br>*Resource includes reference to a systematic review or meta analyses in the body of the resource or in a reference list* | | |
| **4. Is the web resource informed by best practice guidelines?**<br>*Resources includes reference to a best practice guideline in the body of the resource or in a reference list* | | |
| **5. Is the quality of the evidence reported?**<br>*Resource states the quality of evidence; or the website has a site-wide policy which states the quality of the evidence; or the resource is a summary of a larger report which states the quality of the evidence* | | |
| **6. Is the strength of recommendations provided?**<br>*The resource uses GRADE (or GRADE criteria) to inform any recommendations; or the website has a policy which states that GRADE criteria are used to inform any recommendations; or the resource is a summary of a larger report which uses GRADE criteria to inform any recommendations* | | |
| Total – Evidence-base | /6 | |
| Transparency | | |
| **7. Are peer-reviewed sources provided for each claim/recommendation?**<br>*Resource uses in-text citations from credible peer reviewed sources for each claim/recommendation* | | |
| **8. Are the authors' or editors' name and affiliations disclosed?**<br>*The authors' names and affiliations are stated within the resources; or the website provides the name and affiliation of an authority who takes responsibility for the website content* | | |
| **9. Is the advertising clearly labelled (or is there no advertising)?** | | |
| **10. The web resource has been created or updated within the last 3 years?**<br>*The date when the search for evidence was conducted is within 3 years; or the date of the most recent reference in the web resource is within 3 years; or the posting or update date on the web resource is within 3 years* ***(1pt).***<br>*If none of the above (or no date or references within the resource) the resource receives* ***0.5pt*** *if the date of the website is within 3 years.* | | |
| **11. Is there a feedback mechanism?**<br>*Resource includes a 'contact us' link or comments section* | | |
| Total – Transparency | /5 | |

| | | |
|---|---|---|
| Usability | | |
| **12. Logical flow: Is the information easy to follow?**<br>*Resource layout is organized and easy to read; headings/sections are clear and informative* | | |
| **13. Accessibility: Does the web resource offer options to access the information?**<br>*Resource includes text re-size options or screen reader for text content; or resource includes subtitles or a transcription for non-text content* | | |
| Total – Usability | /2 | |

| | |
|---|---|
| **Web Resource Rating Tool score calculation** | |
| ***Step 1 – Evidence-base criteria*** [total score for criteria 1 – 6] ÷ 6 x 75 | /75 |
| ***Step 2 – Transparency & Usability criteria*** [total score for criteria 7 – 13] ÷ 7 x 25 | /25 |
| ***TOTAL SCORE***<br>Total Step 1 + Total Step 2 | /100 |
| ** Note Criteria 10 includes one exception to this individual criteria rating score*<br>*The total web resource rating score is weighted to value content that demonstrates the use of scientific research evidence: the six criteria assessing evidence base comprise 75% of the total score; the seven criteria assessing transparency and usability are combined and comprise 25% of the total score.* | |
